# Comparative evaluation of a new magnetic bead-based DNA extraction method from fecal samples for downstream next-generation 16S rRNA gene sequencing

**DOI:** 10.1371/journal.pone.0202858

**Published:** 2018-08-23

**Authors:** Kara D. McGaughey, Tulay Yilmaz-Swenson, Nourhan M. Elsayed, Dianne A. Cruz, Ramona R. Rodriguez, Michael D. Kritzer, Angel V. Peterchev, Megan Gray, Samantha R. Lewis, Jeffrey Roach, William C. Wetsel, Douglas E. Williamson

**Affiliations:** 1 Department of Psychiatry and Behavioral Sciences, Duke University Medical Center, Durham, North Carolina, United States of America; 2 Mouse Behavioral and Neuroendocrine Analysis Core Facility, Duke University Medical Center, Durham, North Carolina, United States of America; 3 Department of Biomedical Engineering, Duke University, Durham, North Carolina, United States of America; 4 Department of Electrical and Computer Engineering, Duke University, Durham, North Carolina, United States of America; 5 Department of Neurosurgery, Duke University School of Medicine, Durham, North Carolina, United States of America; 6 Promega Corporation, Madison, Wisconsin, United States of America; 7 Research Computing, University of North Carolina at Chapel Hill, Chapel Hill, North Carolina, United States of America; 8 Durham VA Medical Center, Durham, North Carolina, United States of America; Wageningen University, NETHERLANDS

## Abstract

We are colonized by a vast population of genetically diverse microbes, the majority of which are unculturable bacteria that reside within the gastrointestinal tract. As affordable, advanced next-generation sequencing technologies become more widely available, important discoveries about the composition and function of these microbes become increasingly possible. In addition to rapid advancement in sequencing technologies, automated systems have been developed for nucleic acid extraction; however, these methods have yet to be widely used for the isolation of bacterial DNA from fecal samples. Here, we adapted Promega’s Maxwell^®^ RSC PureFood GMO and Authentication kit for use with fecal samples and compared it to the commonly used Qiagen QIAamp^®^ PowerFecal^®^ kit. Results showed that the two approaches yielded similar measures of DNA purity and successful next-generation sequencing amplification and produced comparable composition of microbial communities. However, DNA extraction with the Maxwell^®^ RSC kit produced higher concentrations with a lower fecal sample input weight and took a fraction of the time compared to the QIAamp^®^ PowerFecal^®^ protocol. The results of this study demonstrate that the Promega Maxwell^®^ RSC system can be used for medium-throughput DNA extraction in a time-efficient manner without compromising the quality of the downstream sequencing.

## Introduction

Microbes are found in various niches like the skin, the oral and respiratory tracts, the reproductive tract, and, most abundantly, the gastrointestinal (GI) tract [1]. In humans, the GI tract harbors nearly 100 trillion bacteria that are essential for health, which is more than 10 times the number of human cells in our bodies [2]. However, only a small percentage of these microbes can be cultured from tissue obtained through GI biopsy or fecal sampling [3]. The development of culture-independent methods such as next-generation sequencing (NGS) has enabled rapid advancements in the examination and characterization of microbial communities and their relations with environmental exposure and disease.

Numerous prior studies have shown that the choice of DNA extraction method from fecal samples affects detection of the microbial community structure [4–7]. Fecal samples contain various PCR inhibitors, which can impede the PCR reaction necessary to generate 16S rRNA amplicons prior to sequencing [6]. The presence and concentration of these inhibitors is variable, and the ability of DNA extraction to overcome these substances appears to be dependent upon the extraction protocol. In addition, the first step in DNA extraction—disruption or lysis of bacterial membranes—can bias the capture of specific bacterial taxa due to differences in cell wall structure and integrity [8–10]. Gram-positive bacteria, for example, are characterized by a thick peptidoglycan layer sandwiched between the inner and outer cell membranes. Due to this membrane composition, greater mechanical disruption is required (e.g., bead-beating or heating) to ensure a representative extraction of gram-positive bacterial DNA [7,10]. For this reason, procedures outlined in Qiagen’s QIAamp^®^ PowerFecal^®^ Kit (formally the MO BIO PowerFecal DNA kit; MO BIO Laboratories, Carlsbad, CA), which combines bead-based mechanical disruption with heat exposure have emerged as the preferred method for fecal sample DNA extraction involving downstream NGS [11].

As a result of the technological advancements made in 16S sequencing, there is a parallel opportunity for DNA extraction methods that take advantage of the numerous automated platforms available. Using fecal samples collected from 40 mice, the present study evaluates the performance of the Qiagen QIAamp^®^ PowerFecal^®^ Kit against Promega’s Maxwell^®^ Rapid Sample Concentrator (RSC) PureFood GMO and Authentication Kit, which we modified for fecal samples. Here we compare the extraction time, DNA yield, DNA purity, NGS read quantity and quality, as well as the microbial composition and reproducibility informed by sequencing output between the two methods.

## Materials and methods

### Ethics statement

All animals were handled according to the National Institutes of Health Guide for the Care and Use of the Laboratory Animals and experiments were conducted under an approved protocol by the Duke University Animal Care and Use Committee.

### Sample collection

Forty 8-week old male C57BL/6J mice (Jackson Laboratories, Bar Harbor, ME) were housed in groups of 5 per cage upon arrival. All mice were maintained on a modified reverse 12 hr/12 hr light-dark cycle (lights on 1400 h), with food (ProLab RMH 3500; Purina Labs, Richmond, VA) and water available *ad libitum*. After mice had transitioned into individual housing for downstream behavioral testing, 1–2 g of fecal pellets were collected from cage bedding into a sterile 2 mL microcentrifuge tube using tweezers which were sterilized between subjects. After collection, sample weights were recorded and samples were stored at -80°C until processing.

### DNA extraction

#### Qiagen QIAamp^®^ PowerFecal^®^ Kit

Genomic DNA was isolated from fecal samples using the Qiagen QIAamp^®^ PowerFecal^®^ Kit (Catalog No. 12830–50; Qiagen, Hilden, Germany). DNA extraction was performed following the manufacturer’s instructions with modification to input volume. Input volume was reduced from the recommended 250 mg to prevent overloading the column. Briefly, 150 mg of fecal pellets were added to PowerBead Tubes containing 750 μL of bead solution and vortexed to begin homogenization. Solution C1 was added and the samples were briefly vortexed before incubation at 65°C for 10 min. To aid in collision of the beads with microbial cells and optimize homogenization of the samples, PowerBead Tubes were horizontally secured to an analog vortex mixer using the MO BIO Vortex Adapter (Catalog No. 13000-V1-24; Scientific Industries, Bohemia, NY), shaken for 10 min, and subsequently centrifuged at 10,000 x g for 30 sec. The supernatant was collected and transferred to the provided 2 mL Collection Tube and the remainder of the protocol was followed as recommended by the manufacturer. All samples were eluted in 100 μL of Solution C6, which was left to sit in the spin columns at room temperature for 5 min prior to final centrifugation to maximize DNA yield from the column. This protocol was repeated and DNA was re-extracted for 5 samples, which served as technical replicates to assess kit reproducibility. Extracted DNA was transferred to ThermoFisher Matrix 500 uL screw top tubes and stored at -20°C until downstream application.

#### Maxwell^®^ RSC PureFood GMO and Authentication Kit

As an alternative method, DNA extraction occurred according to a fecal sample-based adaptation to the Maxwell^®^ RSC PureFood GMO and Authentication Kit (Catalog No. AS1600; Promega Corporation, Madison, WI) developed by Promega. Briefly, 75 mg of fecal pellets were placed into a 2 mL microcentrifuge tube, 1 mL of CTAB Buffer was added, and the tubes were vortexed using the MO BIO Vortex Adapter for 30 sec. Following homogenization, samples were heated at 95°C for 5 min and allowed to cool for 2 min on the benchtop before 1 min of thorough horizontal vortex as described above. Samples were then manually homogenized inside 2 mL microcentrifuge tubes using disposable Fisherbrand^™^ Pellet Pestles (Thermo Fisher Scientific, Waltham, MA, USA) until the contents of the fecal pellet were dispersed. To continue lysis, 40 μL of Proteinase K and 20 μL of RNase A were added to the homogenates and samples were horizontally vortexed for 1 min before incubating for 10 min at 70°C. Maxwell^®^ RSC Cartridge preparation and loading occurred as detailed by the manufacturer. All samples were eluted in 100 μL of provided elution buffer. After the run, samples were centrifuged at 12000 x g for 2 min and placed into a magnetic rack for at least 2 min to pellet any remaining magnetic particles before transfer of DNA to ThermoFisher Matrix 500 μL screw top tubes. This protocol was repeated and DNA was re-extracted for 5 samples, which served as technical replicates to assess kit reproducibility. Extracted DNA was stored at -20°C until use in downstream applications.

### Quantification and assessment of purity

For both extraction methods, DNA concentrations were determined fluorometrically using the QuantiFluor^®^ ONE dsDNA System (Promega Corporation, Madison, WI, USA) on a Quantus^®^ fluorometer (Promega Corporation) and purity was assessed via 260/280 and 260/230 absorbance ratios as determined by spectrophotometry (Epoch, Biotek, USA). After processing, samples were stored at -20°C until sequencing.

### Library construction and 16S rRNA gene sequencing

All samples were randomized and normalized to 5 ng/μL with PCR-grade water. Bacterial 16S rRNA amplicons of approximately 460 bp were generated via amplification of the V3 and V4 hypervariable region of the 16S ribosomal RNA gene (16S rRNA) using primer pair sequences purchased from Integrated DNA Technologies, Inc. flanked by Illumina overhang adapter sequences (Forward overhang: 5’TCGTCGGCAGCGTCAGATGTGTATAAGAGACAG. Reverse overhang: 5’GTCTCGTGGGCTCGGAGATGTGTATAAGAGACAG.) In the first round of PCR, the 16S locus was amplified in a 25 μL PCR reaction from a 5 ng/μL template DNA for 25 cycles using 2X KAPA HiFi HotStart ReadyMix (KAPA Biosystems, Wilmington, MA, USA). PCR was performed in a thermal cycler (Biorad C1000 Thermal Cycler, USA) using the following parameter: 95°C^(3:00)^ + [95°C^(0:30)^ + 55°C^(0:30)^ + 72°C^(0:30)^] x 25 cycles + 72°C^(5:00)^ followed by holding at 4°C. The resulting amplicons were purified with AMPure XP beads (Beckman Coulter, High Wycombe, UK) and the expected size (~550 bp) and quality were verified using D1000 Screen Tape (Tapestation 4200; Agilent Technologies, Santa Clara, CA, USA). In the second round of PCR, Illumina sequencing adapters as well as dual-index barcodes were added in a 50 μL PCR reaction from 5 μL of amplicon PCR product for 8 cycles using 2X KAPA HiFi HotStart ReadyMix. PCR was performed in a thermal cycler using the following parameter: 95°C3^(3:00)^ + [95°C^(0:30)^ + 55°C^(0:30)^ + 72°C^(0:30)^] x 8 cycles + 72°C^(5:00)^ followed by holding at 4°C. After index PCR, amplicons were purified with AMPure XP beads and the expected size (~630 bp) and quality of the final library were verified using D1000 Screen Tape. Library concentration was measured using the Qubit^®^ dsDNA HS Assay Kit (Invitrogen, Carlsbad, CA, USA) on a Qubit^®^ 2.0 Fluorometer (Invitrogen, Carlsbad, CA, USA). Purified amplicons were then pooled in equimolar concentrations and mixed with 20% PhiX control library. Sequencing was performed on an Illumina MiSeq (Illumina, San Diego, CA) using the MiSeq v3 reagent kit.

### Bioinformatics analyses

Sequencing output from the Illumina MiSeq platform were converted to fastq format and demultiplexed using Illumina Bcl2Fastq 2.18.0.12 [12]. The resulting paired-end reads were joined using the DADA2 pipeline including merging paired ends, quality filtering, error correction, and chimera detection. Amplicon sequencing units from DADA2 [13] were assigned taxonomic identifiers with respect to the Silva [14] database, their sequences were aligned using template alignment through PyNAST [15], and a phylogenetic tree was built with FastTree 2.1.3 [16]. Quality control of both raw and processed sequencing reads was verified by FastQC [17].

Alpha diversity, with respect to Shannon index, Chao1, and observed species number metrics, was estimated using a rarefaction depth of 100 sequences per subsample. Beta diversity estimates were calculated using weighted and unweighted Unifrac distances [18, 19] between samples at a subsampling depth of 100. Results were summarized and visualized through principal coordinate analysis. Beta diversity values were also used for Procrustes analysis (R package: ‘vegan’) to examine the correspondence between replicate extractions for both kits [20].

### Statistical analysis

Quantitative variables were expressed as mean and standard deviation. Variables were compared using the Fisher’s exact test, Mann-Whitney’s test, Wilcox exact test (R package: ‘exactRankTests’), and linear mixed models using rank transformed data. A *p* value of *p* ≤ 0.05 was considered statistically significant. Analyses were performed using R version 1.0.143 software (RStudio, Inc.) and SPSS version 24.0 software (IBM, Armonk, NY, USA).

## Results

### DNA extraction method impacts DNA yield

**Fig 1:**
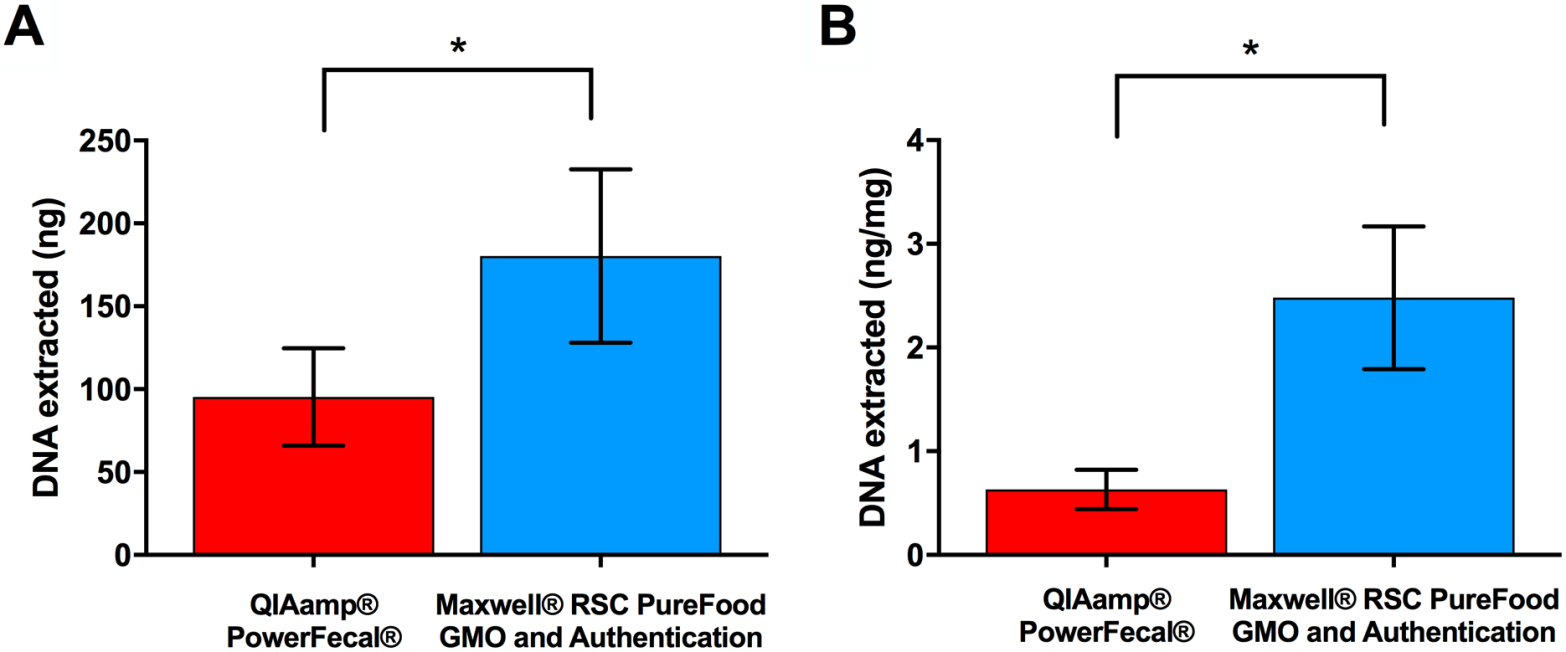
Fecal DNA extraction efficiency varies depending upon extraction method. (A) Mean total amount (± s.d.) of DNA extracted from mouse fecal samples. (B) Mean total amount (± s.d.) of DNA extracted per mg of input material. *n* = 40 individual C57BL/6J mice. Samples were extracted and total DNA was measured by fluorometry. *Statistical significance was determined using a 2-sample *t*-test and *p* ≤ 0.05.

To compare the suitability of Promega’s modified Maxwell^®^ RSC PureFood GMO and Authentication Kit against Qiagen’s QIAamp^®^ PowerFecal^®^ Kit for extraction of bacterial DNA from fecal samples, boli from 40 mice were processed with each method according to the protocols outlined above. The amount of DNA extracted was dependent upon the DNA extraction technique, with the Maxwell^®^ RSC PureFood GMO and Authentication Kit producing greater total DNA yields relative to the QIAamp^®^ PowerFecal^®^ Kit (180.33 ± 52.23 *vs*. 95.40 ± 29.38, *p* ≤ 0.001; Fig 1A). To consider the variability in input material (150 mg for the QIAamp^®^ PowerFecal^®^ Kit *vs*. 75 mg for the Maxwell^®^ RSC PureFood GMO and Authentication Kit), the amount of DNA extracted was normalized by input mass. Despite requiring a lower input mass, the Maxwell^®^ RSC PureFood GMO and Authentication Kit again produced significantly greater yields of total DNA relative to the QIAamp^®^ PowerFecal^®^ Kit (2.48 ± 0.69 *vs*. 0.63 ± 0.19, *p* ≤ 0.001; Fig 1B).

### Extraction method produces comparable DNA quality metrics

Prior to NGS, successful PCR-based amplification requires template DNA containing little to no RNA, proteins, or polysaccharides. The assessment of DNA purity often occurs via spectrophotometry using 260/280 and 260/230 absorbance ratios. DNA samples extracted according to both the QIAamp^®^ PowerFecal^®^ and the Maxwell^®^ RSC PureFood GMO and Authentication kits produced excellent absorbance ratios suggesting relatively pure DNA (Table 1).

**Table 1:** Comparison of DNA extraction methods.

Extraction Method	Manual Time (hr)	Automated Time (hr)	A260/A280 mean ± s.d.	A260/A230 mean ± s.d.	Amplification
**QIAmp**^®^ **PowerFecal**^®^	1.5–2	N/A	1.84 ± 0.02	1.68 ± 0.23	40/40
**Maxwell RSC PureFood GMA Authentication**	0.5	0.6	2.04 ± 0.03	1.86 ± 0.15	39/40

Time of extraction method determined from start of fecal processing to DNA elution and separated into manual and automated components. Mean 260/280 nm and 260/230 nm absorbance ratios (± s.d.) for each extraction method. With respect to more stringent filtering associated with DADA2, successful amplification was defined as minimal coverage of 100 reads per sample. *n* = 40 per extraction method.

### Both extraction methods enable successful amplification and next generation sequencing

Another relevant measure of DNA quality is the performance of extracted DNA samples during downstream applications such as 16S rRNA gene sequencing. Samples were amplified, indexed, and pooled for sequencing on the Illumina MiSeq Platform. With respect to more stringent filtering associated with DADA2 algorithms, which infer sample sequences exactly as opposed to clustering into operational taxonomic units (OTUs), successful amplification was defined as minimal coverage of 100 reads per sample. Notably, the method of DNA extraction had little to no impact on the number of samples that successfully amplified (Table 1). The fact that 40/40 QIAamp^®^ PowerFecal^®^ Kit-extracted and 39/40 Maxwell^®^ RSC PureFood GMO and Authentication Kit-extracted samples amplified above 100 reads suggests that both kits were efficient at overcoming the PCR inhibition associated with fecal samples. After merging, error correction, and filtering with DADA2, QIAamp^®^ PowerFecal^®^ Kit-extracted DNA produced a total of 175,476 high quality sequences with a mean of 4,386.90 ± 3,319 sequences per sample (range: 165–15,148). Maxwell^®^ RSC PureFood GMO and Authentication Kit-extracted DNA produced a total of 202,914 high quality sequences with a mean of 5,072.85 ± 3,059 sequences per sample (range: 16–12,961).

While it is critical that DNA samples can be amplified and sequenced, it is also important that the sequence data are of a high quality for meaningful downstream analysis. NGS platforms, like Illumina, use quality scores (Q), commonly expressed as *Phred* scores that logarithmically relate to base calling error probability. The accepted threshold for base call accuracy is a *Phred* score of 30 (Q30), which is equivalent to the probability of 1 incorrect base call in 1000 calls. Sequences from both QIAamp^®^ PowerFecal^®^- and Maxwell^®^ RSC-extracted DNA resulted in *Phred* scores of 36 and 29 for the forward read and the reverse read, respectively. Following the joining and trimming of paired-end reads, both kits produced *Phred* scores of 37. Collectively, these *Phred* scores suggest that DNA extracted with both the QIAamp^®^ PowerFecal^®^ kit and the Maxwell^®^ RSC PureFood GMO Authentication kit can deliver accurate and usable sequencing data with the Illumina MiSeq platform.

### No kit-based effect of DNA extraction method on microbial diversity

**Fig 2:**
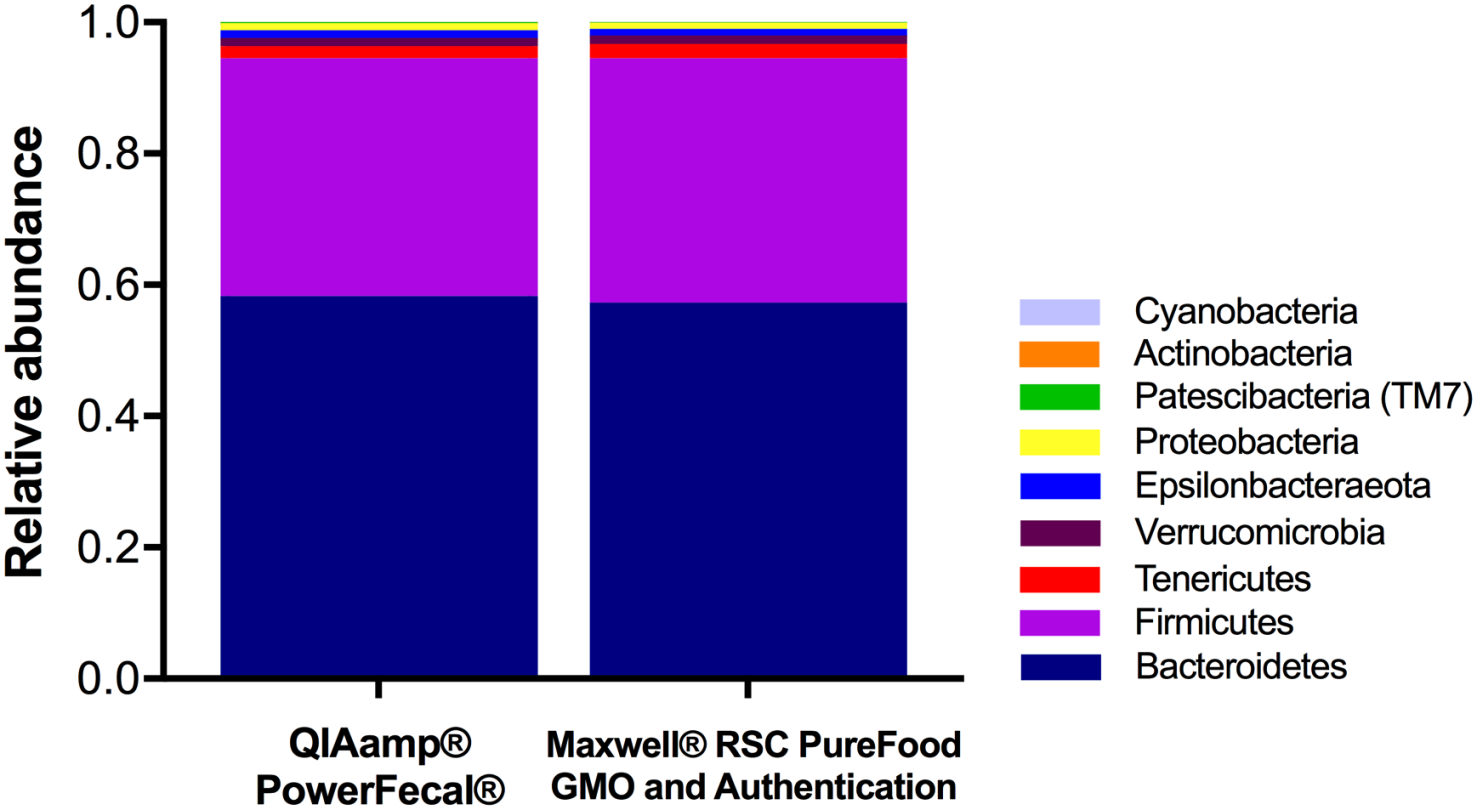
Comparison of DNA extraction methods on next generation sequencing (NGS) relative abundance at the phylum level. NGS using DNA extracted with the QIAamp^®^ PowerFecal^®^ Kit (*n* = 40) and the Maxwell^®^ RSC PureFood GMO and Authentication Kit (*n* = 39) revealed similar proportions of the 9 most prominent phyla. Relative abundances (mean ± s.d.) are available in S1 Table.

**Fig 3:**
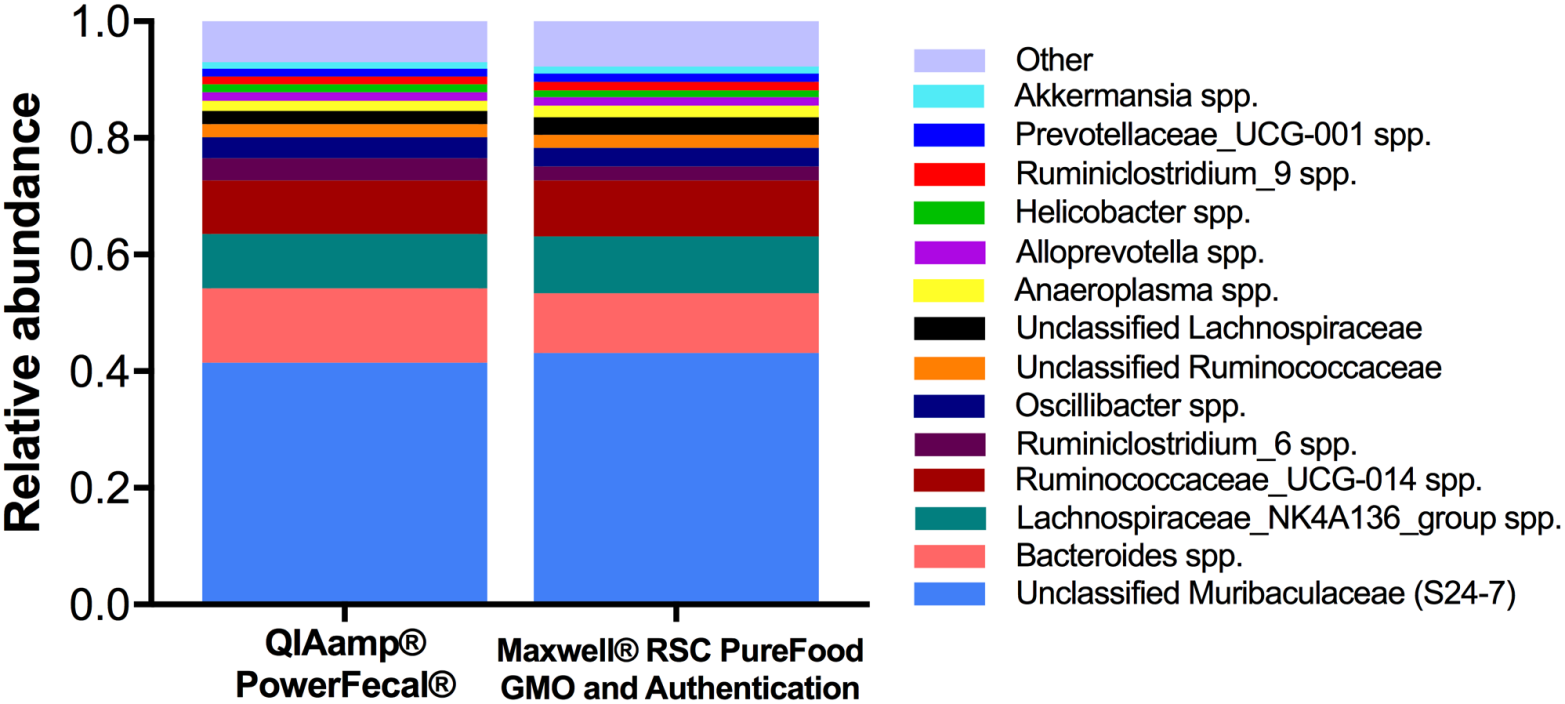
Comparison of DNA extraction methods on next generation sequencing (NGS) relative abundance at the genus level. NGS using DNA extracted with the QIAamp^®^ PowerFecal^®^ Kit (*n* = 40) and the Maxwell^®^ RSC PureFood GMO and Authentication Kit (*n* = 39) revealed similar proportions of most prominent genera. Bacterial genera with a relative abundance ≥ 0.01 (1%) are visualized here. Relative abundances (mean ± s.d.) for the top 30 most abundant genera are available in S2 Table.

To determine whether the extraction method resulted in differential lysis and subsequent skewing of the microbial profile, results of 16S rRNA sequencing were compared. At the phylum level, the Maxwell^®^ RSC PureFood GMO and Authentication Kit-extracted samples and the QIAamp^®^ PowerFecal^®^ Kit-extracted samples revealed essentially the same microbial composition (Fig 2; S1 Table). There were no differences in the relative abundance of major phyla between the two groups (*Bacteroidetes*: QIAamp^®^ PowerFecal^®^ *vs*. Maxwell^®^ RSC: 58.3% *vs*. 57.3%, *p* ≤ 0.52; *Firmicutes*: QIAamp^®^ PowerFecal^®^ *vs*. Maxwell^®^ RSC 36.2% *vs*. 37.3%, *p* ≤ 0.59). To examine the microbial profile resulting from each DNA extraction method kit at a higher taxonomic resolution, relative abundance of genera was compared (Fig 3; S2 Table). Within the top 30 most abundant bacterial genera, the relative abundance of *Lactobacillus* (0.7% *vs*. 0.1%, *p* ≤ 0.001), *Turicibacter* (0.4% *vs*. 0.1%, *p* ≤ 0.05), and several genera within the family *Clostridiales* differed significantly between the QIAamp^®^ PowerFecal^®^ *vs*. Maxwell^®^ RSC kits.

**Fig 4:**
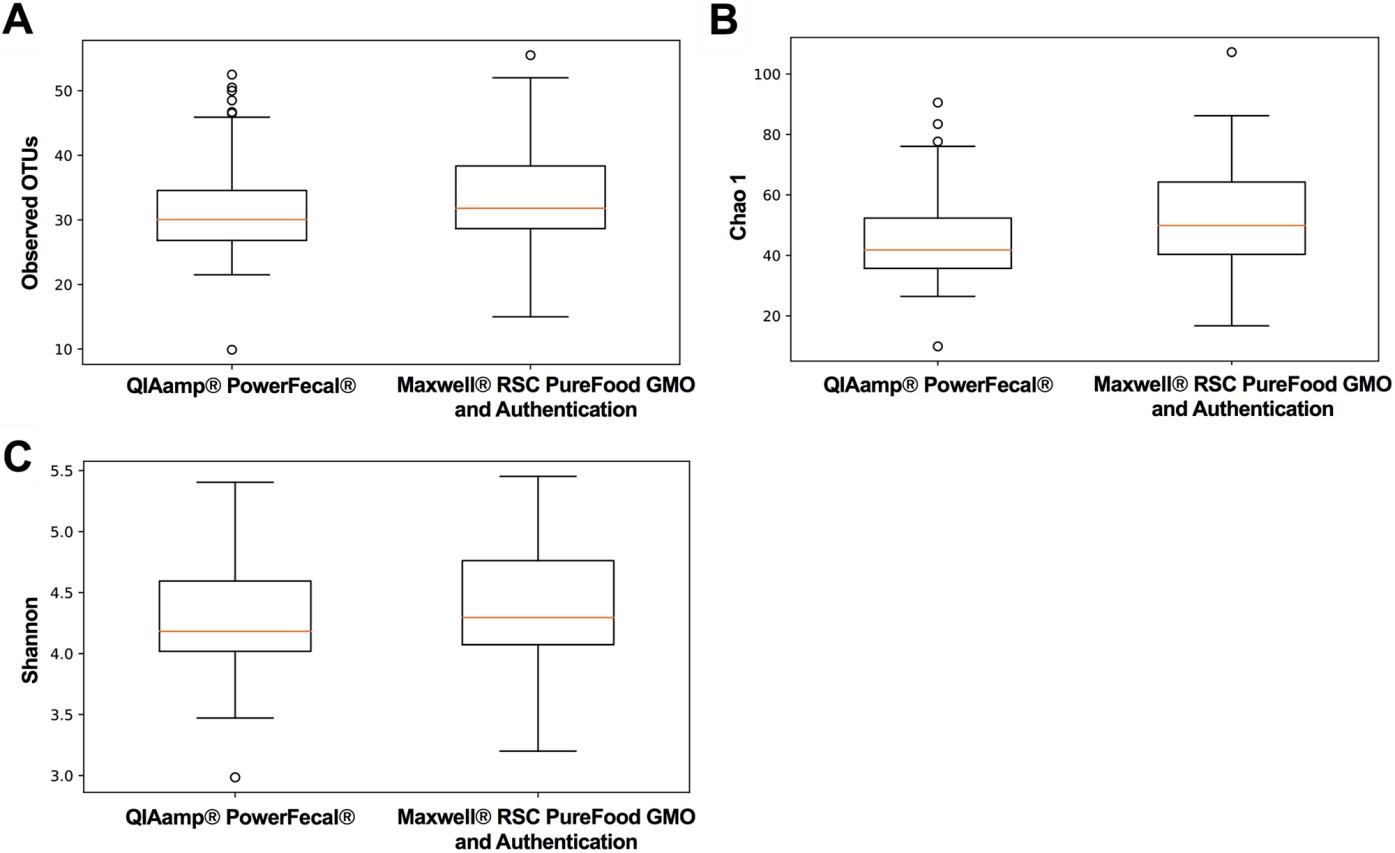
Alpha diversity metrics compared across DNA extraction methods. (A) Box plot comparing number of observed operational taxonomic units (OTUs) across kits. (B) Box plot of Chao1 diversity estimates for the microbial communities in DNA extracted with both the QIAamp^®^ PowerFecal^®^ Kit and the Maxwell^®^ RSC PureFood GMO and Authentication Kit. (C) Box plot of Shannon diversity index comparing the evenness and abundance of species extracted with the two kits.

DNA extracted with the Maxwell^®^ RSC PureFood GMO and Authentication Kit revealed no significant differences in the number of observed OTUs compared to DNA extractions following the QIAamp^®^ PowerFecal^®^ protocol (34.14 ± 9.15 *vs* 32.16 ± 9.13, t = 1.08, *p* ≤ 0.29; Fig 4A). To further examine kit-based differences while accounting for abundance of OTUs, individual *de novo* OTUs were investigated (FDR < 0.01). At this criterion, with 495 unique OTUs identified, only 1 (*Bacteria*; *Firmicutes*; *Clostridia*; *Clostridiales*; *Family*_*XIII*; *Family*_*XIII*_*AD3011*_group; NA) was differentially abundant between the two kits.

As a means of comparing the ability of each extraction method to lyse rare or hard-to-lyse taxa, alpha diversity of samples was also compared using the Chao1 Index (Fig 4B). There was little variability in the Chao1 indices of samples generated via the two DNA extraction methods. While the Maxwell^®^ RSC PureFood GMO and Authentication Kit revealed slightly greater richness, this result was not statistically significant (52.24 ± 17.86 *vs* 46.63 ± 17.18, t = 1.41, *p* ≤ 0.18). Similarly, the Shannon index, another metric of alpha diversity, revealed no significant differences in evenness and abundance between kits (Maxwell^®^ RSC: 4.43 ± 0.54 *vs* QIAamp^®^ PowerFecal^®^: 4.34 ± 0.55, *p* ≤ 0.43; Fig 4C).

**Fig 5:**
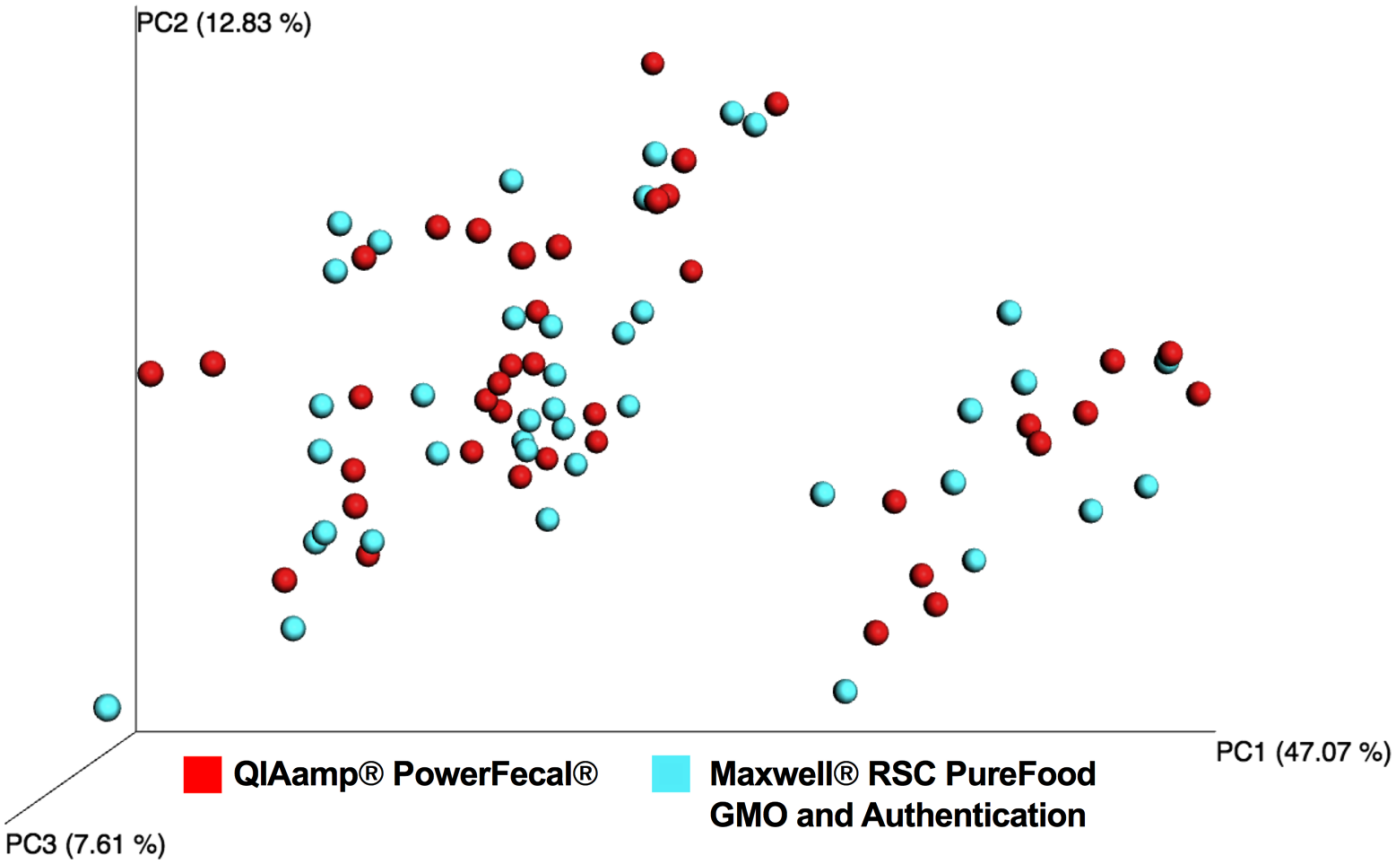
Weighted Unifrac principal coordinate analysis (PCoA) of samples with successful amplification and sequencing. Colors denote DNA extraction method: QIAamp^®^ PowerFecal^®^ Kit (*red*) and Maxwell^®^ RSC PureFood GMO and Authentication Kit (*blue*).

To further evaluate the effect of DNA extraction method on NGS output, principle coordinate analysis (PCoA) was performed (Fig 5). In PCoA, samples that are similar in composition cluster together based on the presence or absence and relative abundance of all OTUs. Analysis of weighted Unifrac PCoA plots using ANOSIM with Monte Carlo Permutation Procedure (MCPP) revealed no clustering or variation as an effect of DNA extraction method (10,000 permutations, non-parametric *p* ≤ 0.35). Individual samples tended to cluster together regardless of which extraction method was used. Results for unweighted Unifrac PCoA analyzed with ANOSIM confirmed this finding (10,000 permutations, non-parametric *p* ≤ 0.24; S1 Fig).

### Reproducibility of DNA extraction methods

Technical replicates were used to assess reproducibility of the QIAamp^®^ PowerFecal^®^ and Maxwell^®^ RSC PureFood GMO and Authentication Kit protocols. Of the initial 40 samples, 5 were chosen at random to be re-extracted with both kits. Replicates were sequenced on the same run as to control for between-sequencing variability.

**Fig 6:**
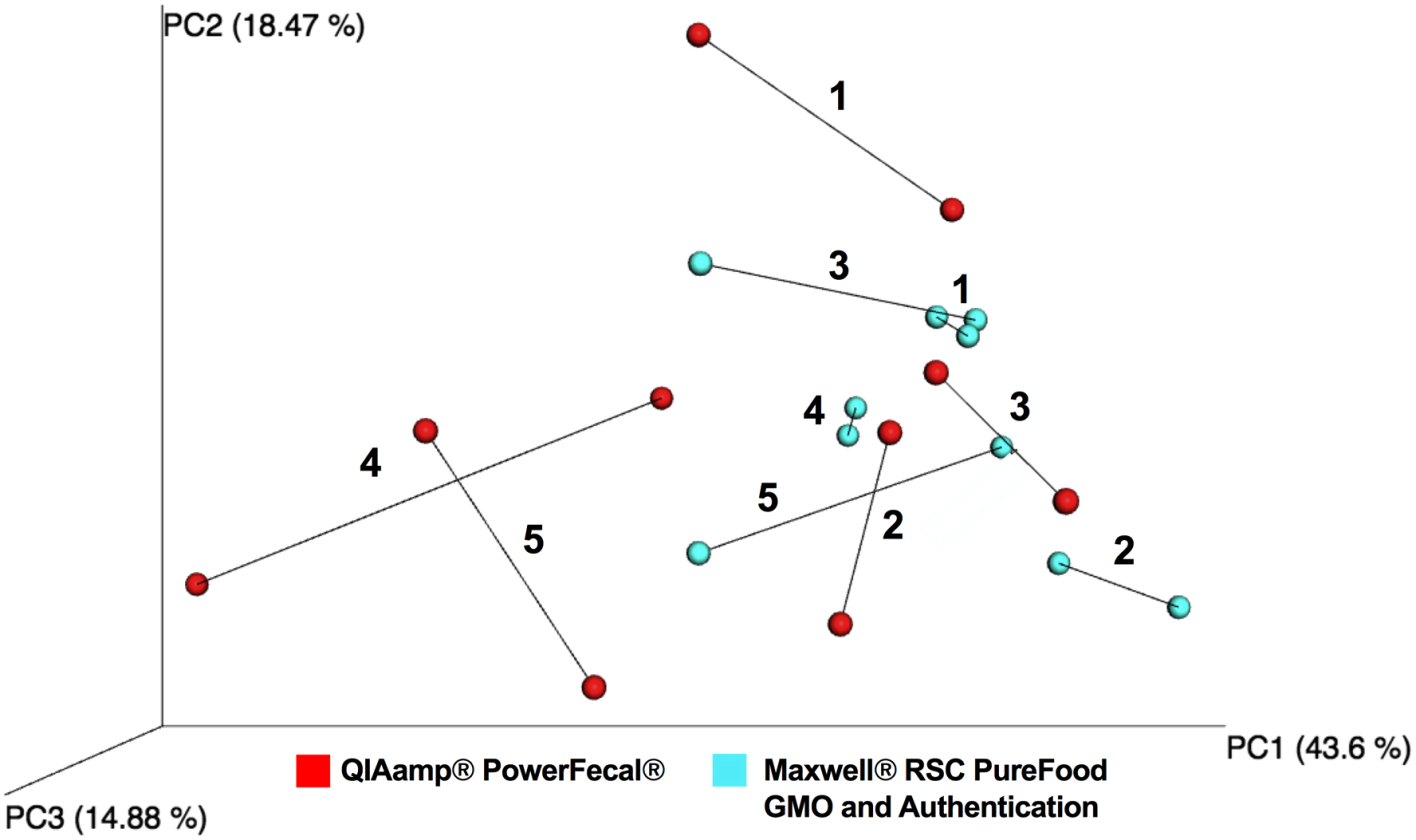
Procrustes-based comparison of microbial composition where longer lines indicate more within-subject dissimilarity of the microbiome. Colors denote DNA extraction method: QIAamp^®^ PowerFecal^®^ Kit (*red*) and Maxwell^®^ RSC PureFood GMO and Authentication Kit (*blue*). Numbers 1–5 denote the 5 technical replicate pairs sequenced with each kit.

Similarity and relatedness of microbial composition between extractions of the same sample was measured using Procrustes analysis. By scaling and superimposing principal coordinate plots, Procrustes enables quantification of non-random congruence between two different measurements from the same group of subjects. The M^2^ statistic produced by the analysis ranges from 0, which signifies that the sample matrices are identical or highly similar, to 1, which implies that sample matrices are completely dissimilar. Procrustes analysis using permutation tests of 16S rRNA gene sequences of the 5 replicate pairs extracted with the QIAamp^®^ PowerFecal^®^ kit (Monte Carlo *p* ≤ 0.075, M^2^ = 0.129) and the 5 replicate pairs extracted with the Maxwell^®^ RSC PureFood GMO and Authentication Kit (Monte Carlo *p* ≤ 0.04, M^2^ = 0.049) revealed stronger similarity between Maxwell^®^ RSC-extracted samples. Procrustes results from both QIAamp^®^ PowerFecal^®^ and Maxwell^®^ RSC PureFood GMO and Authentication Kit technical replicates were overlaid for visual comparison of microbial composition (Fig 6).

**Fig 7:**
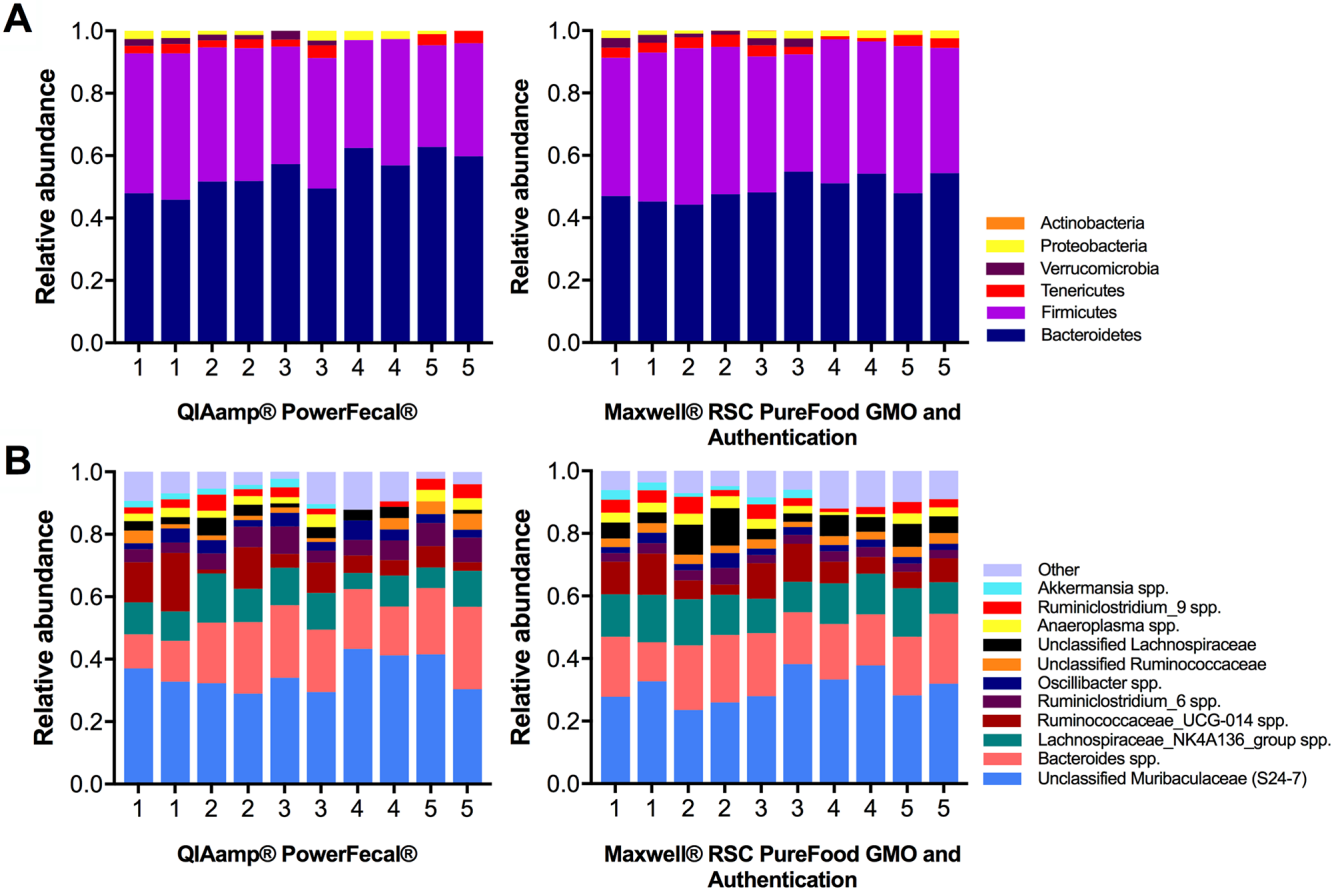
Comparison of relative abundance between DNA technical replicate pairs. (A) Analysis of relative abundance at the phylum level revealed similar proportions of most prominent phyla within technical replicate pairs. (B) Relative abundance of major genera was also similar when compared within and across replicate pairs.

Reproducibility of DNA isolation methodologies was also assessed by comparing the relative abundance of major phyla and genera of replicate pairs across extractions (Fig 7). There were no significant differences between replicate extractions across phylum level taxa for the Maxwell^®^ RSC (F_6,56_ = 0.29, *p* < .942), or for the QIAamp^®^ PowerFecal^®^ kit (F_6,56_ = 1.091, *p* < .379). Similarly, replicate extraction revealed no significant differences at the genus level (Maxwell^®^ RSC kit: F_46,376_ = 0.60, *p* = 0.98; QIAamp^®^ PowerFecal^®^ kit: F_46,376_ = 0.26, *p* < 0.953).

## Discussion

With the decreasing cost of NGS, characterization of microbial communities from fecal samples is becoming increasingly accessible. In the present study, the performance of a novel, modified Maxwell^®^ RSC-based DNA extraction protocol for mouse fecal samples is evaluated against the widely used QIAamp^®^ PowerFecal^®^ Kit. Taken as a whole, data suggest that the two kits produce comparable purity, sequence quality, and representation of microbial composition. Moreover, analysis of technical replicates indicates that both DNA extraction methods are reproducible.

DNA extracted in accordance with both the QIAamp^®^ PowerFecal^®^ and the Maxwell^®^ RSC PureFood GMO and Authentication Kit produced absorbance ratios indicative of relatively pure DNA. The A260/A280 ratio for samples extracted with the Maxwell^®^ RSC PureFood GMO and Authentication Kit was slightly higher than the expected ratio of 1.8 for DNA. High A260/A280 purity ratios do not necessarily present a problem for downstream sequencing and can suggest anything from carryover of magnetic beads to a poor-quality blank eliminating too much signal near the 280nm wavelength. Although purity ratios are an important metric of sample quality, the best indicator is functionality in the downstream application of interest. Importantly, the extracted DNA was successfully amplified in 40/40 samples extracted via the QIAamp^®^ PowerFecal^®^ protocol and 39/40 samples extracted with the Maxwell^®^ RSC PureFood GMO and Authentication Kit, which implies that both kits were efficient at overcoming the PCR inhibition associated with the high polysaccharide content of fecal samples.

Results of 16S rRNA sequencing revealed no significant differences in the relative abundance of *Bacteroidetes* and *Firmicutes*, two of the major phyla which collectively comprise roughly 85% of the mouse gut microbiome. While further examination of taxonomic abundance at the genus level revealed subtle differences in *Lactobacillus*, *Turicibacter*, and several genera within the family *Clostridiaceae*, these taxa comprise only 2.8%, or less, of detected genera. With some genera, like that within the family *S24-7*, constituting 46% of sequences, it is difficult to discern whether or not differences in the abundance of these less prevalent taxa carry biological relevance. Moreover, when individual *de novo* OTUs were compared between kits, only 1 of 495 was significantly differentially abundant. Such small differences in NGS-based characterizations of microbial communities demonstrate the ability of both kits to lyse varying membrane types as well as hard-to-lyse bacterial taxa. It is also important to consider that subtle taxonomic variation could be attributed not only to extraction method differences, but variability in the fecal boli themselves. Analysis of weighted Unifrac PCoA confirmed the similarity of reported microbial composition as samples clustered irrespective of extraction method. To verify that these observations were due to kit-based comparisons and not bioinformatic analysis, we considered QIIME as another pipeline for amplicon sequence variant calling and Greengenes as another database for taxonomic assignment. Results were similar (S3–S6 Tables; S2–S5 Figs).

Collectively, these data illustrate that successful amplification and sequencing are possible with both DNA extraction methods and subsequent NGS microbial profiles are comparable. Not only do the QIAamp^®^ PowerFecal^®^ and Maxwell^®^ RSC PureFood GMO and Authentication kits produce comparable microbial profiles, but, for both kits, this microbial composition was stable across repeated DNA extractions as revealed through examination of technical replicates.

Importantly, while the DNA extracted with the Maxwell^®^ RSC kit produces comparable purity, amplification, sequencing quality, and microbial composition, the Maxwell^®^ RSC protocol requires less manual time per sample (30 min compared to the 1.5–2 hr required by the QIAamp^®^ PowerFecal^®^ Kit). In addition to the ability to process more samples in less time, the Maxwell^®^ RSC PureFood GMO and Authentication Kit produced more of DNA per weight of input material, suggesting more efficient bacterial lysis, as well as an overall increase in the number of high quality sequences. Therefore, in cases where collection of fecal samples is difficult or limited, the Maxwell^®^ RSC PureFood GMO and Authentication Kit permits the extraction of more DNA from less input material. However, since 16S rRNA gene amplification involves the normalization of DNA to a standard volume and concentration, both methods in this study provided sufficient quantity of DNA for normalization and, in turn, sequencing.

It is, however, worth noting that kit comparisons were made using fecal samples collected from C57BL/6J mice. Future investigation is necessary to determine how microbial profiles generated using the Maxwell^®^ RSC-based extraction method compare across host species. As such, researchers should take into account the microbial composition of their ecosystem when considering application of this technology.

In sum, these data reveal that automated systems, like Promega’s Maxwell^®^ RSC magnetic bead-based technology, retain the accuracy of more traditional, more manual extraction methods and can be successfully used for downstream NGS. This automation enables processing of a greater number of samples without compromising integrity and quality, an important factor in scaling-up for higher-throughput experiments.

## Supporting information

S1 Table Relative abundance of bacterial phyla. Relative abundance (mean ± s.d.) of bacterial phyla in samples extracted with QIAamp^®^ PowerFecal^®^ (*n* = 40) and Maxwell^®^ RSC PureFood GMO and Authentication (*n* = 39) kits.
(DOCX)

S2 Table Top 30 most abundant bacterial genera. Relative abundance (mean ± s.d.) of bacterial genera in samples extracted with QIAamp^®^ PowerFecal^®^ (*n* = 40) and Maxwell^®^ RSC PureFood GMO and Authentication (*n* = 39) kits.
(DOCX)

S3 Table Sequencing metrics compared across QIIME and DADA2. While analysis using DADA2 as opposed to QIIME greatly reduced the total number of high quality sequences, the relationship between QIAamp^®^ PowerFecal^®^ and Maxwell^®^ RSC PureFood GMO and Authentication kits remained the same (15% increase in reads with Maxwell^®^ RSC-extracted DNA when analyzed with QIIME *vs*. 15.6% increase in reads with Maxwell^®^ RSC-extracted DNA when analyzed with DADA2).
(DOCX)

S4 Table Relative abundance of bacterial phyla and genera with amplicon sequence variant calling performed using DADA2 and mapping done with Greengenes. As with data analyzed using QIIME, there were no differences in the relative abundance of major phyla between the two groups (*Bacteroidetes*: QIAamp^®^ PowerFecal^®^ *vs*. Maxwell^®^ RSC: 58.3% *vs*. 57.3%, *p* ≤ 0.52; *Firmicutes*: QIAamp^®^ PowerFecal^®^ *vs*. Maxwell^®^ RSC 36.2% *vs*. 37.3%, *p* ≤ 0.59). At the genus level, within the top 30 most abundant bacterial genera, 6 were significantly between the QIAamp^®^ PowerFecal^®^ and Maxwell^®^ RSC kits (*Lactobacillus*: 0.7% *vs*. 0.1%, *p* ≤ 0.000; an unclassified genus within *Clostridiaceae*: 0.4% *vs*. 0.1%, *p* ≤ 0.01; two genera within *Ruminococcaceae*: 0.7% *vs*. 0.4%, *p* ≤ 0.01 and 1.3% *vs*. 1.9%, *p* ≤ 0.01; and two genera within *Lachnospiraceae*: 0.1% *vs*. 0.3%, *p* ≤ 0.01 and 0.1% *vs*. 0.3%, *p* ≤ 0.01). However, investigation of individual *de novo* OTUs (FDR < 0.01) revealed that with 495 unique OTUs identified, only 1 OTU (k_*Bacteria*; p_*Firmicutes*; c_*Clostridia*; o_*Clostridiales*; f_[*Mogibacteriaceae*]; g_; s_) was differentially abundant between the two kits.
(XLSX)

S5 Table Relative abundance of bacterial phyla and genera with amplicon sequence variant calling performed using QIIME and mapping done with Greengenes. There were no differences in the relative abundance of major phyla between the two groups (*Bacteroidetes*: QIAamp^®^ PowerFecal^®^ *vs*. Maxwell^®^ RSC: 55.9% *vs*. 53.9%, *p* ≤ 0.22; *Firmicutes*: QIAamp^®^ PowerFecal^®^ *vs*. Maxwell^®^ RSC 34.4% *vs*. 35.9%, *p* ≤ 0.41). From the top 30 most abundant bacterial genera, 4 were significantly different between the QIAamp^®^ PowerFecal^®^ *vs*. Maxwell^®^ RSC kits (*Lactobacillus*: 0.4% *vs*. 0.1%, *p* ≤ 0.000; an unclassified genus within *Clostridiaceae*: 0.4% *vs*. 0.3%, *p* ≤ 0.01; *Turicibacter*: 0.3% *vs*. 0.1%, p ≤ 0.01; and *Ruminococcus*: 2.7% *vs*. 2.3%, *p* ≤ 0.05). Investigation of individual *de novo* OTUs (FDR < 0.01) revealed that with 25,075 unique OTUs identified, only 2 OTUs (k_*Bacteria*; p_*Firmicutes*; c_*Clostridia*; o_*Clostridiales*; f_*Clostridiaceae*; g_; s_ and k__*Bacteria*; p_*Firmicutes*; c_*Clostridia*; o_*Clostridiales*; f_*Lachnospiraceae*; g_; s_) were differentially abundant between the two kits.
(XLSX)

S6 Table Relative abundance of bacterial phyla and genera with amplicon sequence variant calling performed using QIIME and mapping done with Silva. Similar to data processed through both QIIME and Greengenes as well as DADA2 and Greengenes/Silva, there were no differences in the relative abundance of major phyla between the two groups *Bacteroidetes*: QIAamp^®^ PowerFecal^®^ *vs*. Maxwell^®^ RSC: 55.9% *vs*. 53.9%, *p* ≤ 0.22; *Firmicutes*: QIAamp^®^ PowerFecal^®^ *vs*. Maxwell^®^ RSC 34.5% *vs*. 36.0%, *p* ≤ 0.39). At the genus level, within the top 30 most abundant bacterial genera, 5 differed between the QIAamp^®^ PowerFecal^®^ and Maxwell^®^ RSC kits. Similar to other analyses, this included *Lactobacillus* (0.4% *vs*. 0.1%, *p* ≤ 0.000), an unclassified genus within *Clostridiaceae* (0.4% *vs*. 0.3%, *p* ≤ 0.01), and *Turicibacter* (0.3% *vs*. 0.1%, *p* ≤ 0.01). *Ruminococcus* (2.7% *vs*. 2.3%, *p* ≤ 0.05) and *Allobaculum* (0.1% *vs*. 0.0%, *p* ≤ 0.000) also appeared to differ between extraction methodologies. Investigation of individual *de novo* OTUs (FDR < 0.01) revealed that with 25,136 unique OTUs identified, only 1 (*Bacteria*; *Firmicutes*; *Clostridia*; *Clostridiales*; *Family XIII Incertae Sedis*; uncultured; uncultured bacterium) was differentially abundant between the two kits.
(XLSX)

S1 Fig Unweighted Unifrac principal coordinate analysis (PCoA) of samples with successful amplification and sequencing. Colors denote DNA extraction method: QIAamp^®^ PowerFecal^®^ Kit (*red*) and Maxwell^®^ RSC PureFood GMO and Authentication Kit (*blue*). The four clusters correspond to animals that were co-housed prior to fecal sample collection.
(TIFF)

S2 Fig Alpha diversity DADA2 and Greengenes. As with analysis in QIIME and Greengenes, there were no significant differences in the number of observed OTUs between QIAamp^®^ PowerFecal^®^ and Maxwell^®^ RSC kits (*p* ≤ 0.31). There was also little variability in the Chao1 indices of samples generated via the two DNA extraction methods. While the Maxwell^®^ RSC PureFood GMO and Authentication Kit revealed slightly greater richness, this result was not statistically significant (*p* ≤ 0.24). Similarly, the Shannon index, another metric of alpha diversity, revealed no significant differences in evenness and abundance between kits (*p* ≤ 0.56).
(TIFF)

S3 Fig Alpha diversity QIIME and Greengenes. When observed at a sampling depth of 9,000, DNA extracted with the Maxwell^®^ RSC PureFood GMO and Authentication Kit revealed a greater number of observed operational taxonomic units (OTUs) compared to DNA extractions following the QIAamp^®^ PowerFecal^®^ protocol (*p* ≤ 0.031). This suggests subtle differences in the ability to observe and report overall diversity of microbial communities between the two kits. However, the increase in observed OTUs could stem from the fact that Maxwell^®^ RSC- extracted DNA produced 15% more sequences than DNA extracted with the QIAamp^®^ PowerFecal^®^ protocol.
(TIFF)

S4 Fig Alpha diversity QIIME and Silva. With analysis done using QIIME and mapping done with Silva, there were no significant differences in the number of observed OTUs between QIAamp^®^ PowerFecal^®^ and Maxwell^®^ RSC kits (*p* ≤ 0.29). There was also little variability in the Chao1 indices of samples generated via the two DNA extraction methods. While the Maxwell^®^ RSC PureFood GMO and Authentication Kit revealed slightly greater richness, this result was not statistically significant (*p* ≤ 0.18). Similarly, the Shannon index, another metric of alpha diversity, revealed no significant differences in evenness and abundance between kits (*p* ≤ 0.43).
(TIFF)

S5 Fig Beta diversity. Analysis of weighted Unifrac PCoA plots using ANOSIM with Monte Carlo Permutation Procedure (MCPP) revealed no clustering or variation as an effect of DNA extraction method regardless of which bioinformatics pipeline or which taxonomic assignment database was used: (A) DADA2 and Greengenes: non-parametric *p* ≤ 0.35; (B) QIIME and Greengenes: non-parametric *p* ≤ 0.787; (C) QIIME and Silva: non-parametric *p* ≤ 0.20.
(TIFF)
