# Correction: Comparative evaluation of a new magnetic bead-based DNA extraction method from fecal samples for downstream next-generation 16S rRNA gene sequencing

**DOI:** 10.1371/journal.pone.0212712

**Published:** 2019-02-19

**Authors:** Kara D. McGaughey, Tulay Yilmaz-Swenson, Nourhan M. Elsayed, Dianne A. Cruz, Ramona M. Rodriguez, Michael D. Kritzer, Angel V. Peterchev, Megan Gray, Samantha R. Lewis, Jeffrey Roach, William C. Wetsel, Douglas E. Williamson

The fifth author’s name is spelled incorrectly. The correct name is: Ramona M. Rodriguez. The correct citation is: McGaughey KD, Yilmaz-Swenson T, Elsayed NM, Cruz DA, Rodriguez RM, Kritzer MD, et al. (2018) Comparative evaluation of a new magnetic bead-based DNA extraction method from fecal samples for downstream next-generation 16S rRNA gene sequencing. PLoS ONE 13(8): e0202858. https://doi.org/10.1371/journal.pone.0202858

## References

[1] McGaughey KD, Yilmaz-Swenson T, Elsayed NM, Cruz DA, Rodriguez RR, Kritzer MD, et al (2018) Comparative evaluation of a new magnetic bead-based DNA extraction method from fecal samples for downstream next-generation 16S rRNA gene sequencing. PLoS ONE 13(8): e0202858 10.1371/journal.pone.0202858 30138447 PMC6107275

