# Supplementary material for: Comparative evaluation of a new magnetic bead-based DNA extraction method from fecal samples for downstream next-generation 16S rRNA gene sequencing
Source: PLoS One. 2018 Aug 23;13(8):e0202858. doi: 10.1371/journal.pone.0202858 (PMC6107275; doi:10.1371/journal.pone.0202858)
Supplement: S1 Table — Relative abundance (mean ± s.d.) of bacterial phyla in samples extracted with QIAamp® PowerFecal® (n = 40) and Maxwell® RSC PureFood GMO and Authentication (n = 39) kits. (DOCX) [file pone.0202858.s001.docx]

|  | **QIAamp® PowerFecal®** | **Maxwell® RSC** | ***p* value** |
| --- | --- | --- | --- |
| *Actinobacteria* | 0.13 ± 0.31 | 0.02 ± 0.06 | 1.19E-02 |
| *Proteobacteria* | 0.83 ± 0.77 | 1.00 ± 0.85 | 3.20E-01 |
| *Cyanobacteria* | 0.02 ± 0.07 | 0.03 ± 0.12 | 4.22E-01 |
| *Bacteroidetes* | 58.31 ± 11.00 | 57.26 ± 9.36 | 5.23E-01 |
| *Verrucomicrobia* | 0.96 ± 0.78 | 1.10 ± 0.89 | 5.66E-01 |
| *Firmicutes* | 36.21 ± 12.29 | 37.31 ± 10.08 | 5.88E-01 |
| *Patescibacteria* | 0.30 ± 0.74 | 0.27 ± 0.56 | 7.69E-01 |
| *Tenericutes* | 1.87 ± 1.67 | 2.10 ± 2.19 | 8.26E-01 |
| *Epsilonbacteraeota* | 1.36 ± 2.85 | 0.91 ± 1.81 | 8.75E-01 |
| *NA;NA* | 0.00 ± 0.01 | 0.00 ± 0.00 | 1 |
