# Supplementary material for: Comparative evaluation of a new magnetic bead-based DNA extraction method from fecal samples for downstream next-generation 16S rRNA gene sequencing
Source: PLoS One. 2018 Aug 23;13(8):e0202858. doi: 10.1371/journal.pone.0202858 (PMC6107275; doi:10.1371/journal.pone.0202858)
Supplement: S2 Table — Relative abundance (mean ± s.d.) of bacterial genera in samples extracted with QIAamp® PowerFecal® (n = 40) and Maxwell® RSC PureFood GMO and Authentication (n = 39) kits. (DOCX) [file pone.0202858.s002.docx]

|  | **QIAamp® PowerFecal®** | **Maxwell® RSC** | ***p* value** |
| --- | --- | --- | --- |
| *Lactobacillus* spp. | 0.66 ± 0.69 | 0.11 ± 0.11 | 5.54E-08 |
| *Ruminiclostridium*_5 spp. | 0.42 ± 0.37 | 0.97 ± 0.98 | 5.84E-05 |
| *Clostridium_sensu_stricto_1* spp. | 0.37 ± 0.43 | 0.08 ± 0.08 | 8.39E-04 |
| Unclassified *Lachnospiraceae* | 2.20 ± 1.04 | 3.04 ± 3.01 | 5.68E-03 |
| *Ruminiclostridium_6* spp. | 3.82 ± 2.64 | 2.44 ± 2.47 | 9.24E-03 |
| *Lachnoclostridium* spp. | 0.11 ± 0.19 | 0.31 ± 0.30 | 2.21E-02 |
| *Turicibacter* spp. | 0.44 ± 0.64 | 0.14 ± 0.15 | 3.82E-02 |
| *Bifidobacterium* spp. | 0.12 ± 0.31 | 0.02 ± 0.02 | 4.80E-02 |
| *Ruminococcus_1* spp. | 0.34 ± 0.83 | 0.40 ± 0.41 | 6.95E-02 |
| *Oscillibacter* spp. | 3.57 ± 1.34 | 3.17 ± 3.17 | 1.33E-01 |
| *Ruminiclostridium_9* spp. | 1.31 ± 1.30 | 1.74 ± 1.78 | 1.39E-01 |
| *Ruminiclostridium* spp. | 0.79 ± 0.79 | 0.92 ± 0.93 | 2.58E-01 |
| Unclassified *Clostridiales_vadinBB60_group* | 0.43 ± 0.35 | 0.54 ± 0.53 | 2.96E-01 |
| Unclassified *Mollicutes_RF39* | 0.11 ± 0.13 | 0.08 ± 0.08 | 3.10E-01 |
| *Bacteroides* spp. | 12.70 ± 10.57 | 10.36 ± 10.44 | 3.56E-01 |
| *Parasutterella* spp. | 0.76 ± 0.71 | 0.88 ± 0.84 | 4.21E-01 |
| *Ruminococcaceae_UCG-014* spp. | 9.19 ± 7.38 | 9.53 ± 9.75 | 5.16E-01 |
| Unclassified *Muribaculaceae* | 41.57 ± 11.54 | 43.12 ± 42.82 | 5.49E-01 |
| *Akkermansia* spp. | 0.96 ± 0.78 | 1.10 ± 1.13 | 5.66E-01 |
| Unclassified *Ruminococcaceae* | 2.28 ± 1.07 | 2.22 ± 2.27 | 5.73E-01 |
| *Muribaculum* spp. | 0.33 ± 0.60 | 0.26 ± 0.25 | 7.51E-01 |
| *Candidatus_Saccharimonas* spp. | 0.30 ± 0.74 | 0.27 ± 0.24 | 7.69E-01 |
| *Anaeroplasma* spp. | 1.76 ± 1.65 | 2.02 ± 2.07 | 7.81E-01 |
| *Parabacteroides* spp. | 0.34 ± 0.75 | 0.24 ± 0.23 | 7.87E-01 |
| *Prevotellaceae_UCG-001* spp. | 1.29 ± 2.61 | 1.33 ± 1.23 | 8.65E-01 |
| *Helicobacter* spp. | 1.36 ± 2.85 | 0.91 ± 0.79 | 8.75E-01 |
| *Lachnospiraceae_NK4A136_group* spp. | 9.34 ± 3.30 | 9.75 ± 9.83 | 9.11E-01 |
| *Alistipes* spp. | 0.20 ± 0.37 | 0.18 ± 0.18 | 9.37E-01 |
| *Alloprevotella* spp. | 1.44 ± 2.98 | 1.40 ± 1.40 | 9.71E-01 |
| *Paraprevotella* spp. | 0.27 ± 0.80 | 0.25 ± 0.25 | 9.81E-01 |
