# Supplementary material for: Comparative evaluation of a new magnetic bead-based DNA extraction method from fecal samples for downstream next-generation 16S rRNA gene sequencing
Source: PLoS One. 2018 Aug 23;13(8):e0202858. doi: 10.1371/journal.pone.0202858 (PMC6107275; doi:10.1371/journal.pone.0202858)
Supplement: S3 Table — While analysis using DADA2 as opposed to QIIME greatly reduced the total number of high quality sequences, the relationship between QIAamp® PowerFecal® and Maxwell® RSC PureFood GMO and Authentication kits remained the same (15% increase in reads with Maxwell® RSC-extracted DNA when analyzed with QIIME vs. 15.6% increase in reads with Maxwell® RSC-extracted DNA when analyzed with DADA2). (DOCX) [file pone.0202858.s003.docx]

|  | **Kit** | **Number of sequences** | **Mean ± s.d.** | **Range** |
| --- | --- | --- | --- | --- |
| QIIME | QIAamp® PowerFecal® | 1,833,386 | 45,834.65 ± 28,448 | 9,664 – 134,304 |
|  | Maxwell® RSC | 2,109,190 | 52,729.75 ± 28,755 | 1,151 – 119,157 |
| DADA2 | QIAamp® PowerFecal® | 175,476 | 4,386.90 ± 3,319 | 165 – 15,148 |
|  | Maxwell® RSC | 202,914 | 5,072.85 ± 3,059 | 16 – 12,961 |
|  |  |  |  |  |
|  |  |  |  |  |
