# Supplementary figures and images for: Comparative evaluation of a new magnetic bead-based DNA extraction method from fecal samples for downstream next-generation 16S rRNA gene sequencing
Source: PLoS One. 2018 Aug 23;13(8):e0202858. doi: 10.1371/journal.pone.0202858 (PMC6107275; doi:10.1371/journal.pone.0202858)

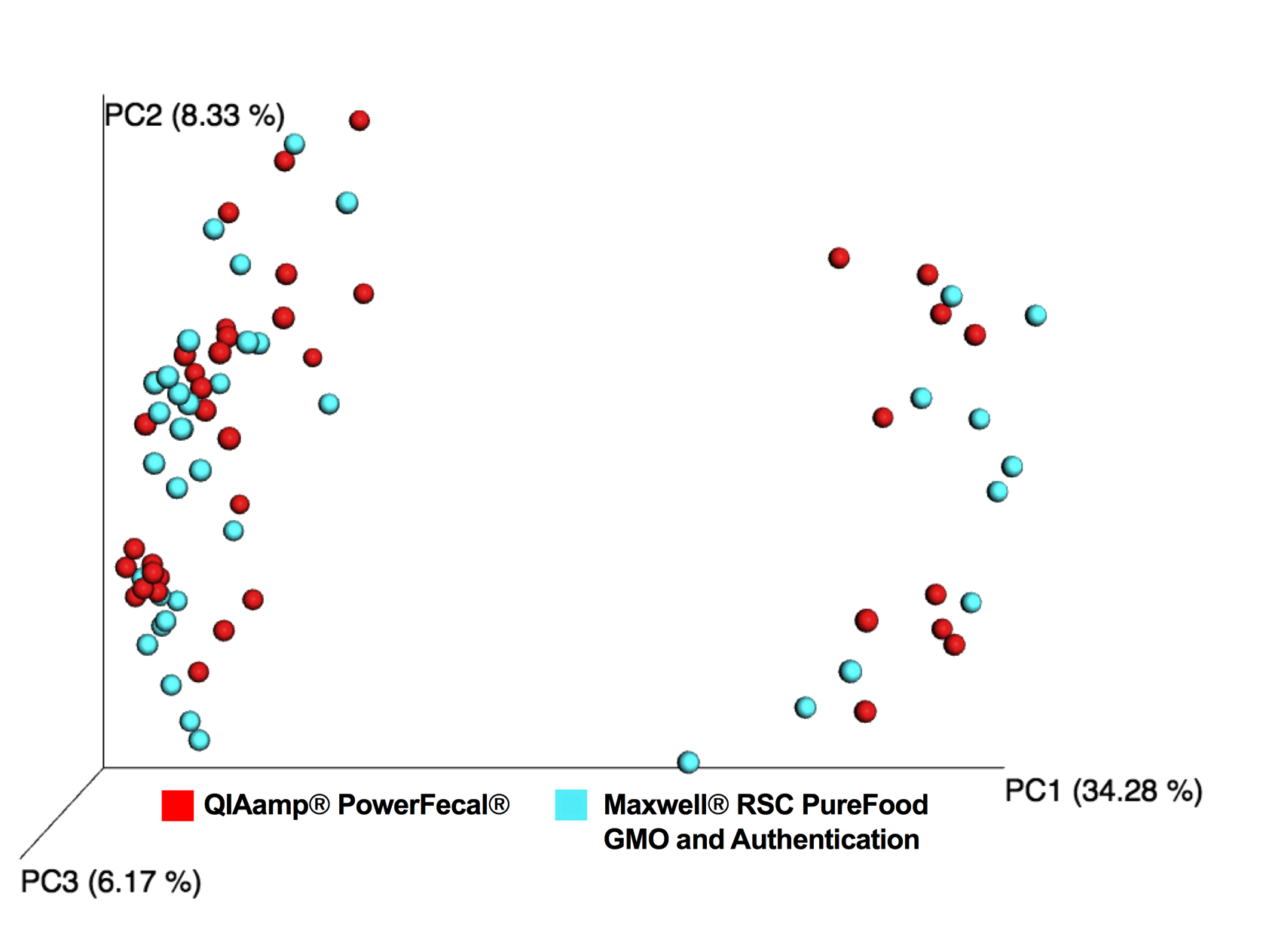

Supplement: S1 Fig — Colors denote DNA extraction method: QIAamp® PowerFecal® Kit (red) and Maxwell® RSC PureFood GMO and Authentication Kit (blue). The four clusters correspond to animals that were co-housed prior to fecal sample collection. (TIFF) [file pone.0202858.s007.tiff]

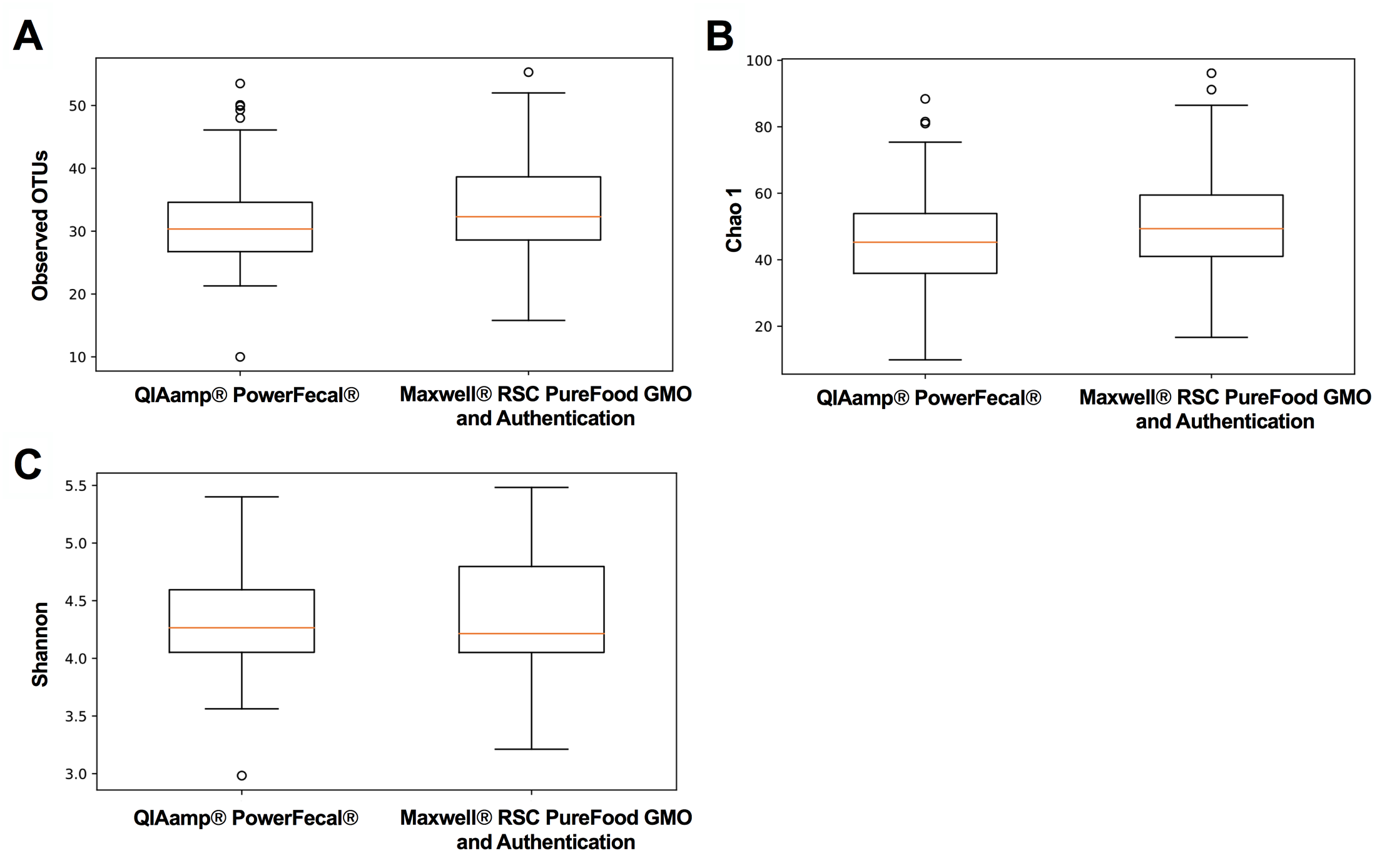

Supplement: S2 Fig — As with analysis in QIIME and Greengenes, there were no significant differences in the number of observed OTUs between QIAamp® PowerFecal® and Maxwell® RSC kits (p ≤ 0.31). There was also little variability in the Chao1 indices of samples generated via the two DNA extraction methods. While the Maxwell® RSC PureFood GMO and Authentication Kit revealed slightly greater richness, this result was not statistically significant (p ≤ 0.24). Similarly, the Shannon index, another metric of alpha diversity, revealed no significant differences in evenness and abundance between kits (p ≤ 0.56). (TIFF) [file pone.0202858.s008.tiff]

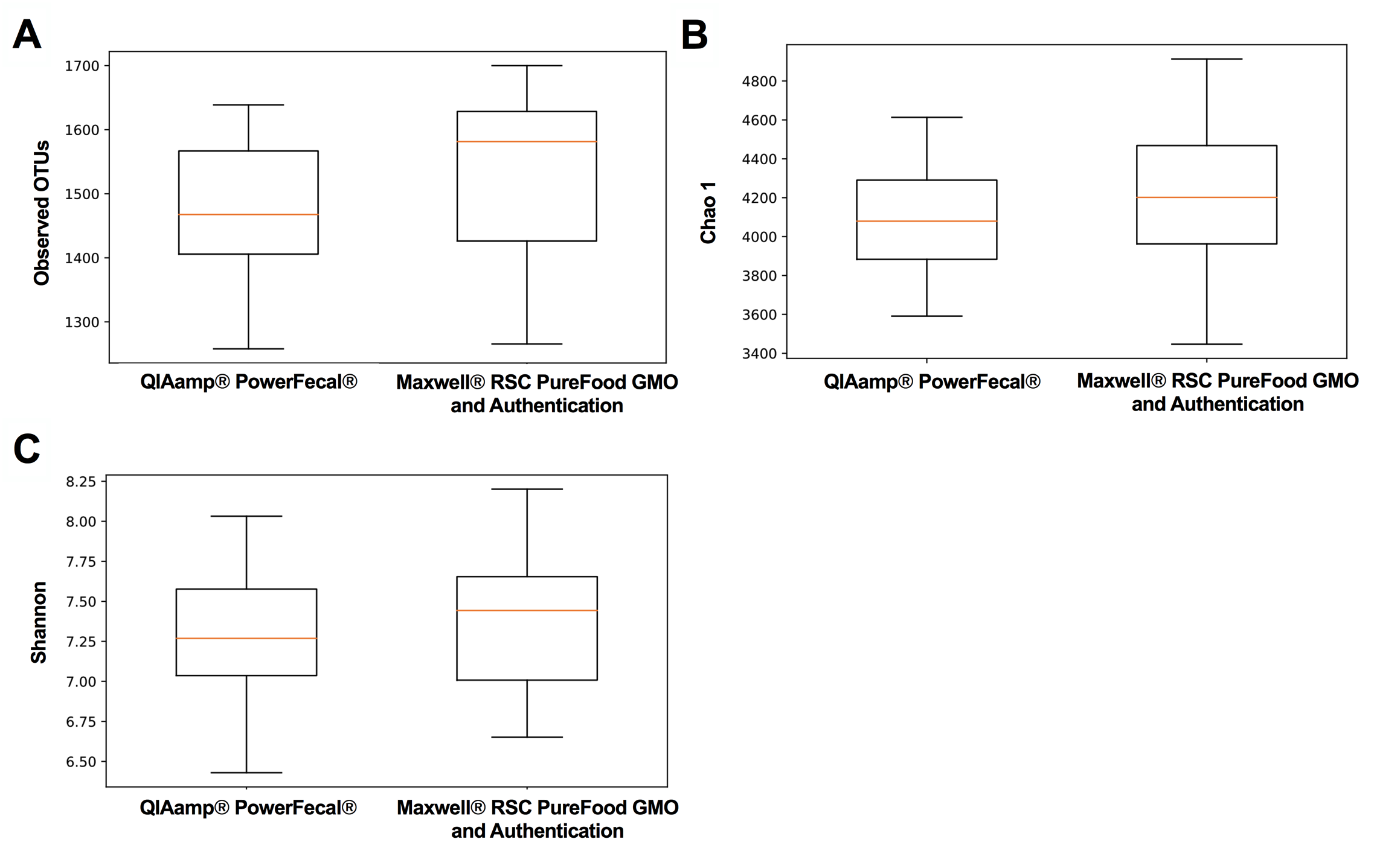

Supplement: S3 Fig — When observed at a sampling depth of 9,000, DNA extracted with the Maxwell® RSC PureFood GMO and Authentication Kit revealed a greater number of observed operational taxonomic units (OTUs) compared to DNA extractions following the QIAamp® PowerFecal® protocol (p ≤ 0.031). This suggests subtle differences in the ability to observe and report overall diversity of microbial communities between the two kits. However, the increase in observed OTUs could stem from the fact that Maxwell® RSC- extracted DNA produced 15% more sequences than DNA extracted with the QIAamp® PowerFecal® protocol. (TIFF) [file pone.0202858.s009.tiff]

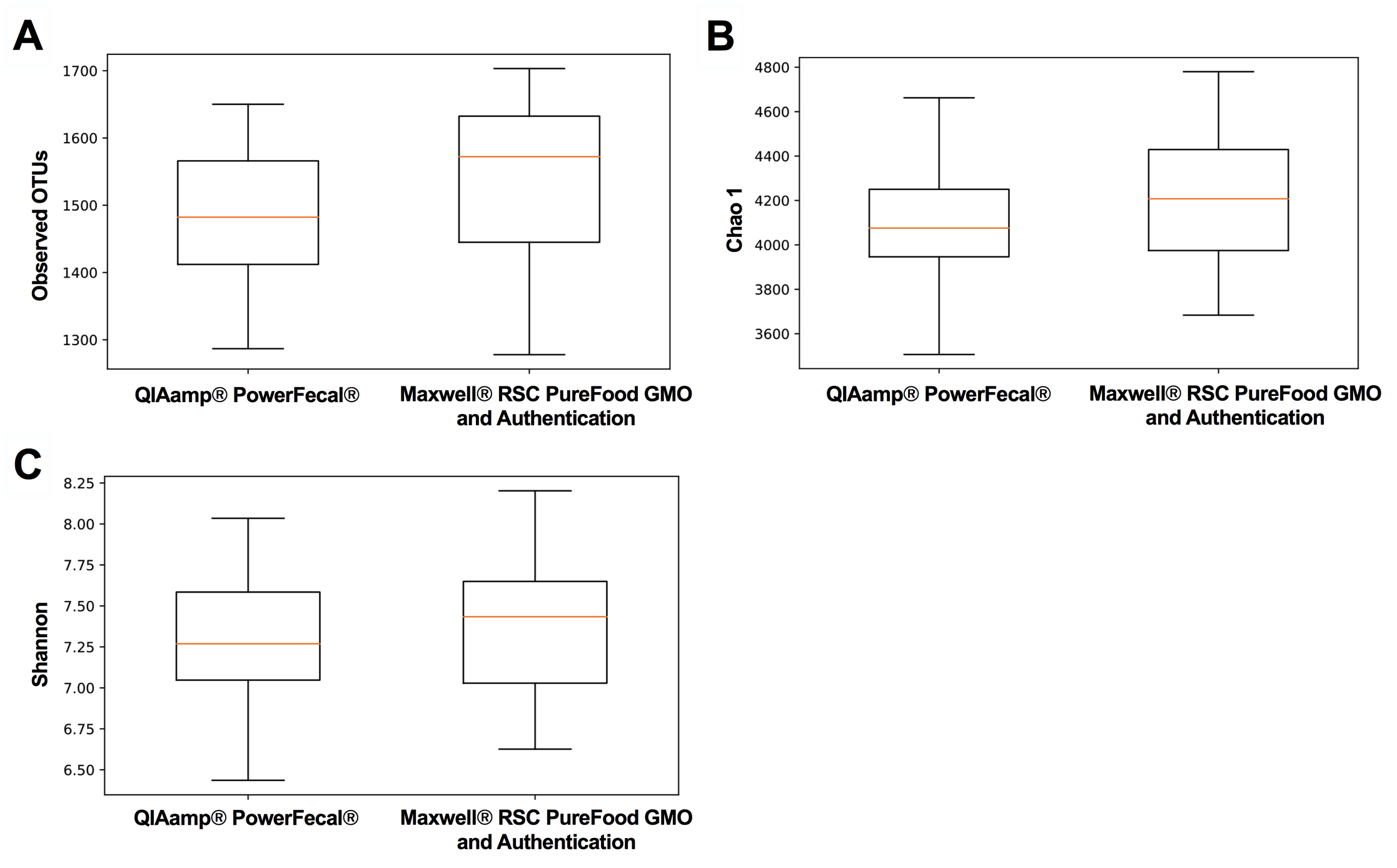

Supplement: S4 Fig — With analysis done using QIIME and mapping done with Silva, there were no significant differences in the number of observed OTUs between QIAamp® PowerFecal® and Maxwell® RSC kits (p ≤ 0.29). There was also little variability in the Chao1 indices of samples generated via the two DNA extraction methods. While the Maxwell® RSC PureFood GMO and Authentication Kit revealed slightly greater richness, this result was not statistically significant (p ≤ 0.18). Similarly, the Shannon index, another metric of alpha diversity, revealed no significant differences in evenness and abundance between kits (p ≤ 0.43). (TIFF) [file pone.0202858.s010.tiff]

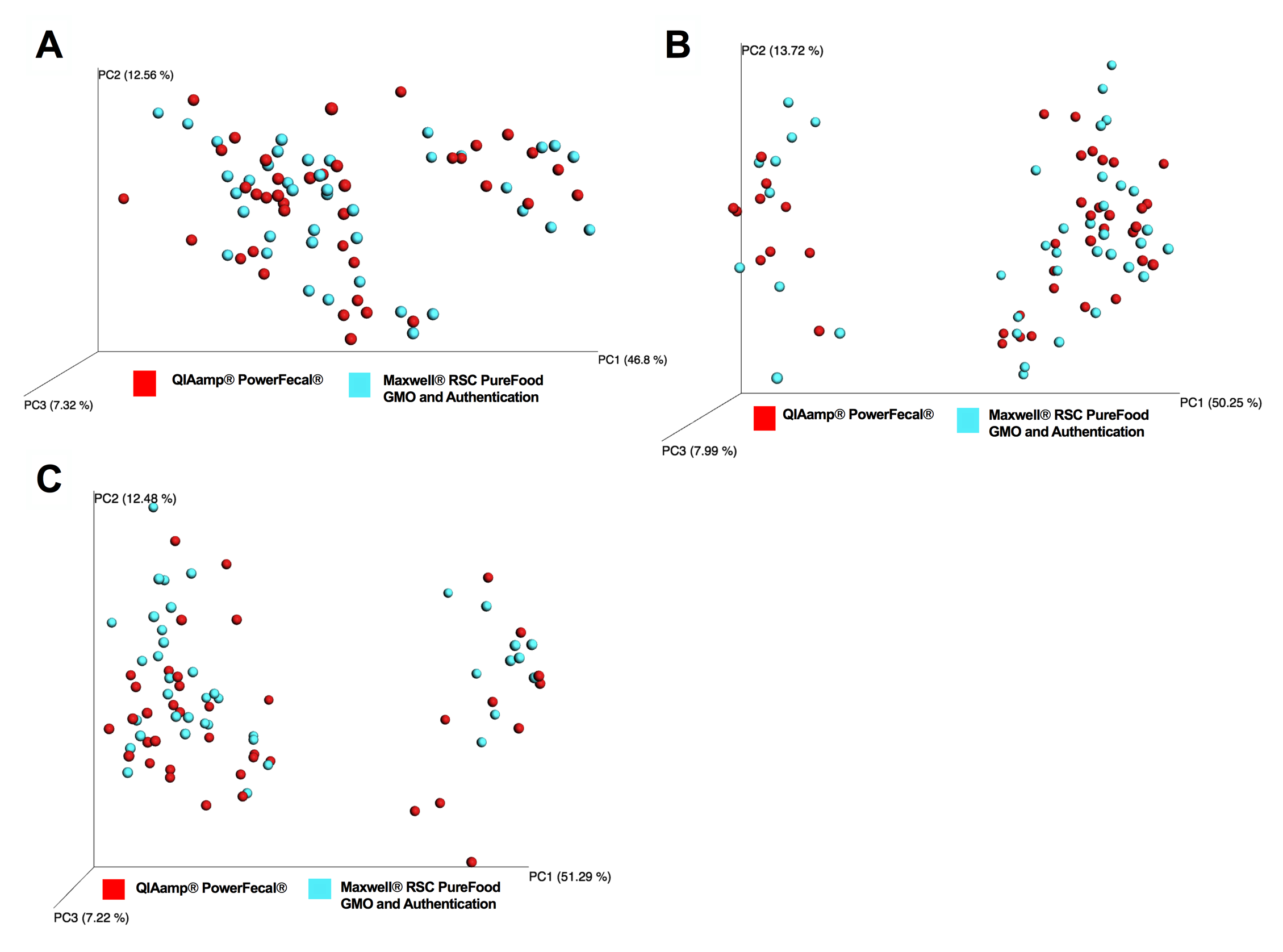

Supplement: S5 Fig — Analysis of weighted Unifrac PCoA plots using ANOSIM with Monte Carlo Permutation Procedure (MCPP) revealed no clustering or variation as an effect of DNA extraction method regardless of which bioinformatics pipeline or which taxonomic assignment database was used: (A) DADA2 and Greengenes: non-parametric p ≤ 0.35; (B) QIIME and Greengenes: non-parametric p ≤ 0.787; (C) QIIME and Silva: non-parametric p ≤ 0.20. (TIFF) [file pone.0202858.s011.tiff]
